# Improvement in Fatigue Behavior of Dental Implant Fixtures by Changing Internal Connection Design: An In Vitro Pilot Study

**DOI:** 10.3390/ma12193264

**Published:** 2019-10-07

**Authors:** Nak-Hyun Choi, Hyung-In Yoon, Tae-Hyung Kim, Eun-Jin Park

**Affiliations:** 1 Department of Prosthodontics, School of Medicine, Ewha Womans University, Seoul 07985, Korea; hyun_bc@naver.com; 2 Department of Prosthodontics, School of Dentistry and Dental Research Institute, Seoul National University, Seoul 03080, Korea; prosyhi@naver.com; 3 Kim and Lee Dental Clinic, Seoul 06626, Korea; kthrock@nate.com

**Keywords:** dental implants, fracture strength, mechanical stress, fatigue, dental implant–abutment connection, dental implant–abutment design

## Abstract

(1) Background: The stability of the dental implant–abutment complex is necessary to minimize mechanical complications. The purpose of this study was to compare the behaviors of two internal connection type fixtures, manufactured by the same company, with different connection designs. (2) Methods: 15 implant–abutment complexes were prepared for each group of Osseospeed^®^ TX (TX) and Osseospeed^®^ EV (EV): 3 for single-load fracture tests and 12 for cyclic-loaded fatigue tests (nominal peak values as 80%, 60%, 50%, and 40% of the maximum breaking load) according to international standards (UNI EN ISO 14801:2013). They were assessed with micro-computed tomography (CT), and failure modes were analyzed by scanning electron microscope (SEM) images. (3) Results: The maximum breaking load [TX: 711 ± 36 N (95% CI; 670–752), EV: 791 ± 58 N (95% CI; 725–857)] and fatigue limit (TX: 285 N, EV: 316 N) were higher in EV than those in TX. There was no statistical difference in the fracture areas (*P* > 0.99). All specimens with 40% nominal peak value survived 5 × 10^6^ cycles, while 50% specimens failed before 10^5^ cycles. (4) Conclusions: EV has improved mechanical properties compared with TX. A loading regimen with a nominal peak value between 40% and 50% is ideal for future tests of implant cyclic loading.

## 1. Introduction

Dental implants have been a fairly reliable and predictable treatment option for edentulous patients since their introduction [1]. In previous systematic reviews, a 5-year survival rate of 95.6–97.2% and 10-year survival rate of 93.1% in implants supporting fixed partial dentures were reported, implying that implants have a high survival rate [2,3,4]. Although dental implants have been clinically and scientifically studied as a viable treatment option to restore the edentulous area [5,6,7], complications remain a big concern for clinicians. Complication of dental implants can be mainly classified as either biological or mechanical. Biological complications include early loss of osseointegration, marginal bone loss, and peri-implantitis, eventually leading to the implants failing and falling out. Mechanical complications include loose abutment or screw, veneer or ceramic fracture, loss of retention, sinking down of abutment, and fracture of implant fixture, abutment, or screw [2]. Previous reports have demonstrated incidence rates of implant fixture fracture of 0.2–1.1% and abutment or screw fracture of 0.7–2.3% [2]. In particular, fracture of implant fixture is a catastrophic complication, which requires extensive surgical treatments. To overcome mechanical failure of dental implants and guarantee long-term clinical success, the stability of the implant–abutment connection to withstand a masticatory load is important [4,8]. The mechanical stability of the connection may be affected by modifying the implant–abutment connection design as well as by improving material properties of the components [9]. Recently, manufacturers of dental implant systems such as Astra Tech Dental have introduced a modified connection design of the dental implant fixture to improve its mechanical properties. To date, however, only a few studies have demonstrated the effect of different connection designs on the mechanical properties of the implant fixtures [10].

Fatigue is the process of localized, permanent structural change of a material under fluctuating stress [11]. Mechanical complications of implants are generally caused by fatigue stress related to mechanical overload [12]. The interpretation of fatigue limit in implants is slightly different from general mechanics. The fatigue limit of dental implants is defined as “the maximum loading value that can withstand 5 × 10^6^ cycles”, contrary to the general definition in mechanics: “the maximum loading value that can withstand infinite cycles” [11,13]. To evaluate fatigue stress in the laboratory, finite element analysis and cyclic loading can be utilized [14,15,16]. While finite element analysis is considered to simulate fairly reliable results, cyclic loading is used as a method to observe the mechanical properties of actual specimens [16]. To standardize the testing method in the laboratory, ISO 14801 was suggested to simulate a “worst-case scenario” applied on an implant–abutment assembly and consists of sinusoidally curved cyclic loading [13]. These methods can be utilized to substitute in vivo tests, and while a generalized clinical conclusion may not be drawn, a tendency can be observed to provide insight to researchers and clinicians.

The purpose of this study was to compare the mechanical behaviors of two internal connection type dental implant fixtures with different connection designs manufactured by a single manufacturer. By strictly adhering to the procedures considered as the norm, it may provide data on implant systems widely used on the market, and moreover, supply background for additional protocols to the universal standard. The null hypothesis was that the fatigue behavior including the mode of failure of the dental implant–abutment complex is not affected by the modification of the connection design of the dental implant fixture.

## 2. Materials and Methods

### 2.1. Preparation of Specimens

**Figure 1:**
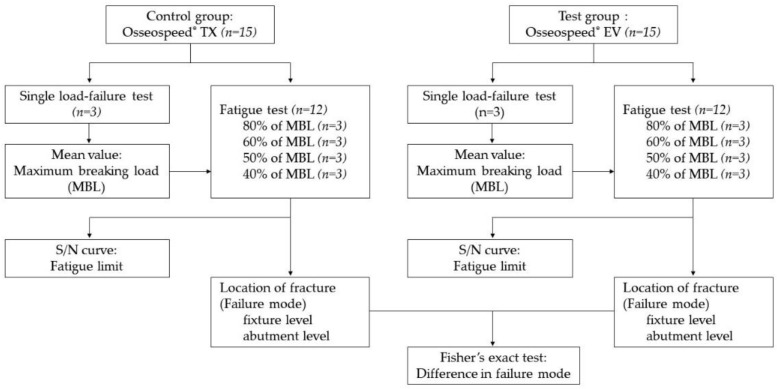
Overall flow of the experiment.

The fixtures and abutments tested in this study are listed in Table 1 and the flow of the experiment is shown in Figure 1. A total of 30 implant–abutment assemblies were prepared for test and control groups (n = 15 per group): Osseospeed^®^ EV (EV) and Osseospeed^®^ TX (TX) (Astra Tech Dental, Dentsply Sirona Implants, Mӧlndal, Sweden). Among the 15 specimens, 3 were tested for single-load fracture tests to identify the maximum fracture load and the other 12 specimens were divided into 4 groups (n = 3 each) for fatigue test under cyclic loading. Each specimen was marked with an indelible marker indicating where the load would be applied to analyze the fractured surface with a scanning electron microscope (SEM). Each implant and abutment was connected with a torque of 25 N cm, as recommended by the manufacturer. 

### 2.2. Micro-CT Image Observation

The implant–abutment assemblies were scanned with micro-computed tomography (CT) scanner (SkyScan 1172, Bruker, Kontich, Belgium) to obtain a series of detailed structural images prior to cyclic loading. The samples were firmly fixed to a full 360° rotational inspection jig. The frame rate was four frames per rotational step of 0.5°, for a total of 2880 images per specimen.

### 2.3. Single-Load Failure Test and Fatigue

**Figure 2:**
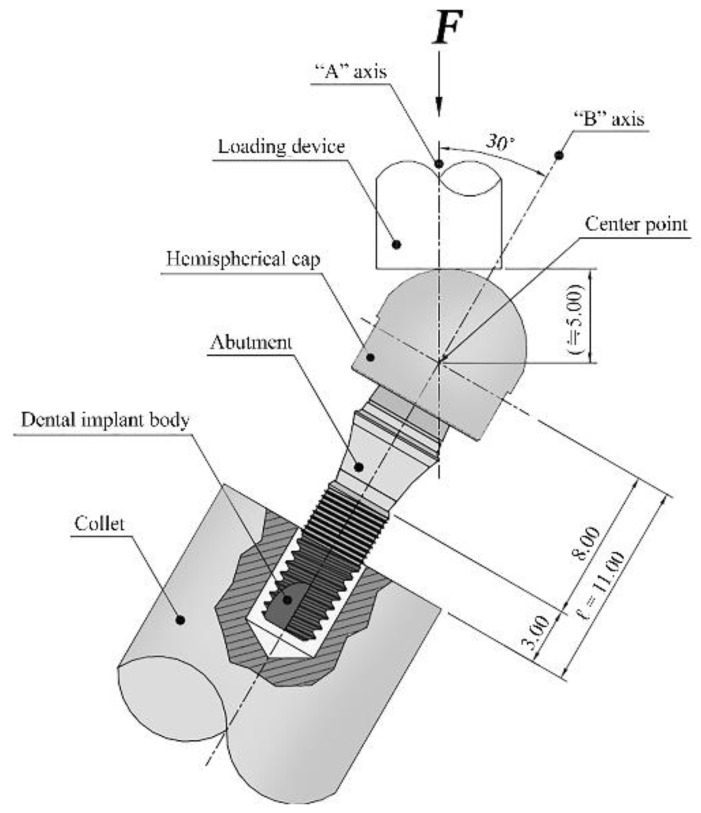
Schematic diagram of the loading test device according to ISO 14801:2013.

All mechanical tests were performed according to ISO 14801:2013 (Figure 2). The testing apparatus should impose a force within ± 5% of the maximum error range of the nominal peak value with constant frequency. The testing apparatus should also be able to monitor maximum and minimum load values and to stop when the specimen fractures. A servo-hydraulic test system (MTS Landmark, Minneapolis, MN, USA) under load control was used. Single-load failure tests and fatigue tests were conducted in an atmospheric environment of 20 °C ± 5 °C. Each implant–abutment assembly was inserted in a custom stainless-steel jig and collet up to the first thread of the implant fixture (approximately 3.0 mm). The collets were then held at a 30° off-axis angle and fixed to the jig and testing machine. A hemispherical cap was engaged to the implant–abutment assembly, contacting the flat head of the universal testing machine. Compressive load increasing at a speed of 1mm/min was applied to the implant–abutment assembly until fracture or deformation occurred. Three implants from each group were tested and their maximum fracture load values were recorded. The average value of maximum fracture load of the tested implants served as the nominal peak value for the fatigue test.

For the fatigue testing under cyclic loading, we applied a sinusoidal oscillation with 15 Hz frequency between a nominal peak level (maximum) and a 10% value of the nominal peak level (minimum) to the implant–abutment assembly. The cyclic loading was conducted until the fracture occurred. If fracture did not occur, the cyclic loading was conducted up to a maximum number of 5 × 10^6^ cycles. The nominal peak levels of 80%, 60%, 50%, and 40% of the maximum fracture load from the previous single-load-to-failure test were selected. Three samples for each nominal peak level group were tested and the number of cycles in which fracture occurred was recorded. If the implant–abutment assembly survived the entire loading cycle, 5 × 10^6^ cycles were recorded. The results were then plotted on an S/N curve, which is a plot of the magnitude of an alternating stress versus the number of cycles to failure for a given material. The S/N curves were estimated by a logarithmic linear regression model utilizing the least squares method. The fatigue limit of the tested dental implant was defined as the maximum fracture load value which can withstand 5 × 10^6^ cycles [13].

### 2.4. Failure Modes and Microscopic Observation

The fractured area of each implant–abutment assembly was microscopically observed and divided into three categories of failures: fixture-level, abutment-level, and screw-level. Two representative specimens were randomly selected before fatigue testing to examine the connection area of the intact implant–abutment assembly using a field emission scanning electron microscope (SEM) (S-4700, Hitachi, Tokyo, Japan). The specimens were inspected one more time after fatigue testing. The frontal and coronal sectional views of fractured specimens were microscopically examined with 15.0 kV accelerating voltage at ×25 and ×30. For the frontal view, specimens were aligned to show the loading direction from left to right. The abutment and fixture cross-sectional views were symmetrically aligned such that the loading direction could be observed from 12 o’clock and 6 o’clock respectively.

### 2.5. Statistical Analysis

Mean values and standard deviations of the maximum breaking loads and mean values of the performed cycle from the fatigue tests were calculated. Fisher’s exact test was used to analyze the numbers of each type of failure to evaluate the difference of failure modes (fixture-level, abutment-level, and screw-level failure). All statistical analyses were performed using SAS^®^ version 9.4 (SAS Institute, Cary, NC, USA). 

## 3. Results

### 3.1. Micro-CT Image Observation

**Figure 3:**
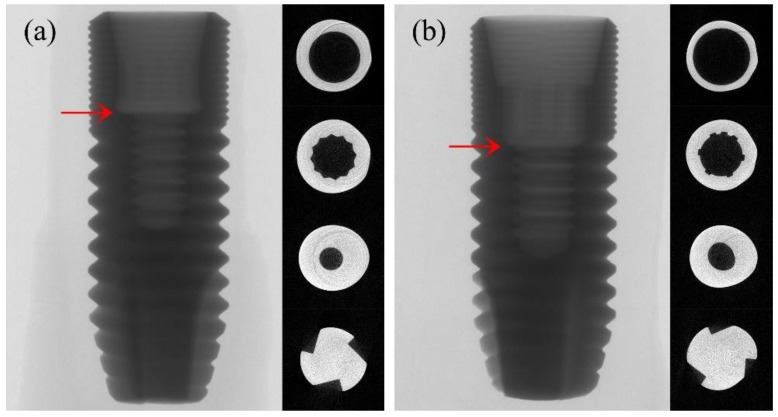
Frontal and cross-sectional micro-CT view: (**a**) TX, (**b**) EV; red arrow = location of the thinnest part of the implant fixture.

Frontal and coronal cross-sectional views of micro-CT showed the detailed design of TX and EV (Figure 3). The thinnest areas, excluding the most coronal portion of the fixture, were expected to be the initiation point of the crack; however, the initiation point was the first thread under the microthread, which does not coincide with the thinnest part. 

### 3.2. Maximum Breaking Load and Fatigue Limit

**Figure 4:**
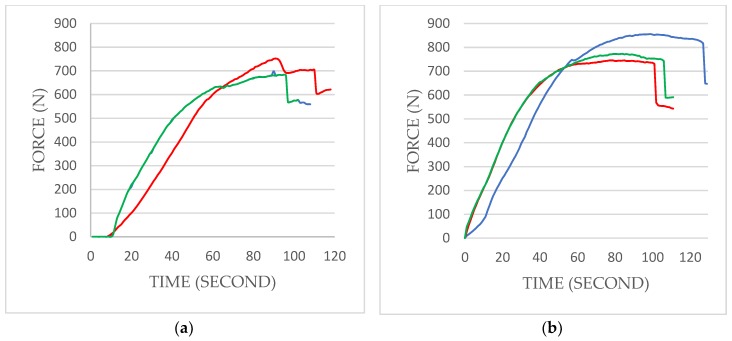
Single-load-to-failure test results with two different implant fixtures: (**a**) TX, (**b**) EV. Compressive load increasing at a speed of 1mm/min was applied. The peak indicates when deformation starts to occur on the implant–abutment assembly, which is the maximum breaking load. The average maximum breaking load of TX = 711 ± 36 N; EV = 791 ± 58 N.

**Figure 5:**
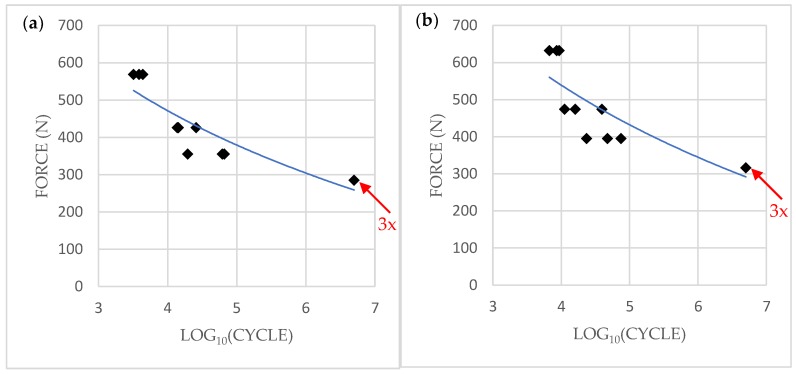
Plotted S/N curves from cyclic loading tests results: (**a**) TX, (**b**) EV. The x-axis represents the logarithmic value of the number of cycles performed. The loading level represents the maximum of the sinusoidal loading level; red arrow = 3 dots overlapped.

The TX samples that underwent single-load failure tests showed a mean maximum breaking load of 711 ± 36 N (95% CI; 670–752), and the EV samples showed an average value of 791 ± 58 N (95% CI; 725–857) (Table 2). The trend of the load and fracture of the specimens was plotted on a time–load diagram, the peak being the point when deformation occurs on the implant–abutment complex (Figure 4). Fatigue testing results are shown in Table 3 and were plotted on an S/N curve with the logarithmic values of the cycles endured on the X-axis and nominal peak level on the Y-axis (Figure 5). All three TX samples of 40% nominal peak level of 285 N endured 5 × 10^6^ cycles, whereas the other nine specimens failed to resist breaking. The fatigue limit was 285 N to withstand 5 × 10^6^ cycles. However, all three EV samples of 40% nominal peak level of 316 N endured 5 × 10^6^ cycles, while the other nine samples failed. The fatigue limit was 316 N to withstand 5 × 10^6^ cycles.

### 3.3. Failure Modes

Failure modes were observed to speculate the fracture mechanism of the TX and EV samples and are shown in Table 4. For the TX groups, every tested assembly except one, which showed abutment-level failure at the 80% loading level, exhibited failure at the fixture level. For the EV groups, two tested assemblies appeared to have torn-out fixtures at the 80% loading level, which were designated as fixture-level failures. The other assemblies exhibited fixture-level fractures occurring between the first and second threads. All fractures of the specimens were accompanied by screw fractures. There was no statistical difference between fractured areas between the TX and EV groups (*P* > 0.99).

### 3.4. Microscopic Observation

**Figure 6:**
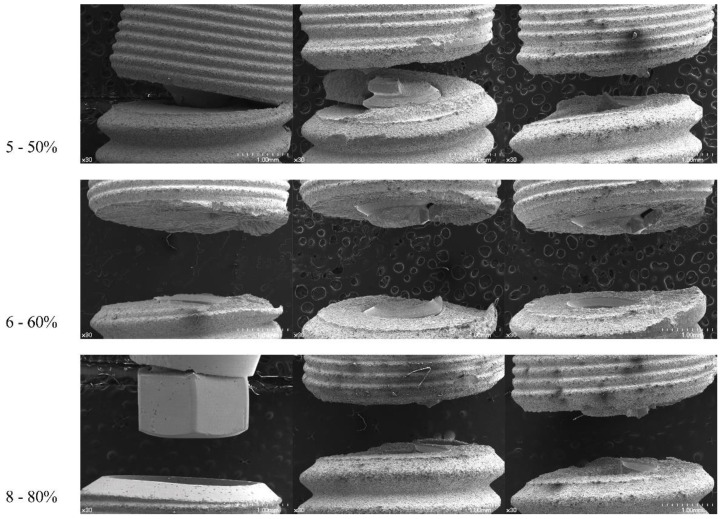
Frontal view of TX samples (×30). Fixtures are aligned to represent a load subjected from left to right.

**Figure 7:**
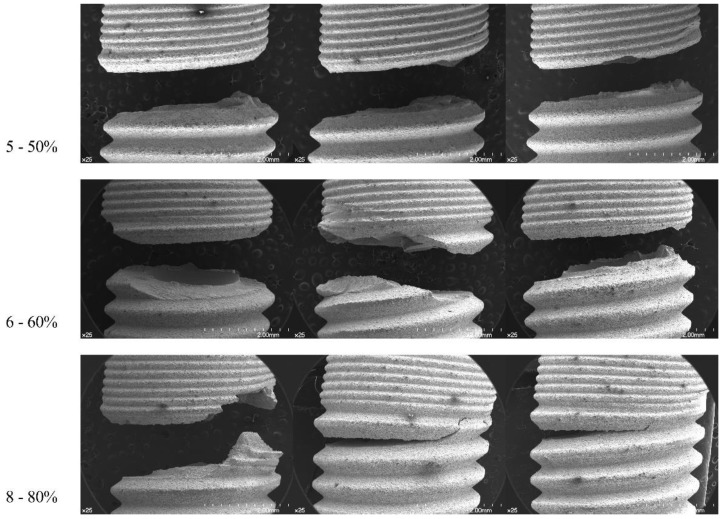
Frontal view of EV samples (×25). Fixtures are aligned to represent a load subjected from left to right.

Based on the SEM examination, all the samples, except one TX sample which had an abutment-level fracture, showed a tendency of fixture-level fracture around the first and second threads apical to the microthread area. The thinnest part at the implant–abutment interface and the fractured area did not correspond for TX specimens (Figure 6). For the EV specimens, the 50% loading-level group was characterized with a clean-cut fracture tendency at the first thread level. The other groups showed a tendency to be torn out in a wavy pattern apical and coronal to the first thread. The fractured area was almost at the same level as the thinnest part of the fixture itself (Figure 7). Therefore, from the results, the null hypothesis was accepted.

## 4. Discussion

During masticatory function, the dental implant fixture and abutment complex should withstand high axial and lateral force of the jaw [17]. An average value of the axial direction force on a single molar implant restoration was previously reported as 120 N [18]. The reported values of maximum loads ranged from 108 to 299 N in the incisor region and from 216 to 847 N in the molar region [18,19,20,21]. In previous research, Park et al. have reported fracture strength under static loading between 799 and 1255 N in the grade 4 titanium implant–abutment assemblies with a diameter close to 4.0 mm [22]. Marchetti et al. have reported fracture strength of 430 N and a fatigue limit of 172 N (i.e., 40% of the maximum breaking load) in a grade 4 titanium implant fixture with a diameter of 3.8 mm [23]. Although a direct comparison between current findings and previous results was impossible due to the difference in the loading conditions between the studies, a similar tendency could be observed. Both TX and EV systems used in this study could overcome the normative requirements, and could be characterized by stable mechanical properties. Furthermore, the calculated fatigue strength proportion between TX and EV in our study was approximately 11%. A study conducted by Johansson and Hellqvist has previously reported that the EV system had 11–20% superior fatigue resistance compared to the TX system, which was consistent with the current findings [24]. The increased strength of EV may be the result of a more apically-located implant–abutment joint area, leading to a better stress distribution, which can be speculated from the micro-CT images. Even with similar chemical compositions, the geometry of the implant–abutment connection can affect the mechanical performance in dental implants. Therefore, the clinician should consider the mechanical properties of implant systems in the treatment planning phase, especially in locations where intraoral conditions may be harsh.

Although ISO 14801 provides a standardized protocol for cyclic loading, it does not provide a loading regimen other than starting at a nominal peak value of 80%, and so one must design the interval between the loading values. This leaves the researcher to guess a nominal peak value that can withstand 5 × 10^6^ cycles, which in this case was 40%. However, a 50% nominal peak value seems to be too high a value to accurately estimate the fatigue limit. The 40% groups of TX and EV in this study that endured 5 × 10^6^ cycles are equivalent to 20 years of service time in the mouth. Previous studies have shown that humans have an average of 250,000 mastication cycles per year [25,26]. Therefore, it can be assumed that 5 × 10^6^ cycles are equivalent to 20 years of service time in the mouth. As the worst-case scenario simulates the harshest environment, it can also be assumed that both specimens can successfully survive intraoral clinical conditions. In contrast, the 50% groups fractured before an average of 50,000 cycles, which is equivalent to less than three months of service time. This “extreme” loading could have affected the failure modes as well as the estimated fatigue limit. Therefore, a loading regimen that includes a nominal peak value between 40% and 50% is recommended for future implant cyclic loading tests. In addition, we speculate that extrapolation to a clinical situation of extreme loading is less applicable for the interpretation of the 50% peak value.

The observation of fractured areas is also important in understanding the fracture mechanism in dental implants. With the advance of technology, micro-CT can be used in observing possible deformations of the dental implant [27]. In this study, the micro-CT images taken before the loading test revealed design differences between the two fixtures. The thinnest part, excluding the coronal portion of the fixture, of TX was located at the microthread area, which was approximately 0.5–1 mm coronal to the first thread of the implant fixture. The thinnest part of EV was located at the first thread of the implant fixture. The thinnest areas of each fixture are shown in Figure 4 and were expected to be the mechanically weakest parts, eventually being the fracture-prone area. However, the fracture lines initiated around the first thread of the fixture in this study. The first thread area was at the same level as the thinnest part in the EV fixture, while they were not at the same level in the TX. This suggested that the weakest part, not necessarily the thinnest part, of the TX and EV fixtures was located around the first thread area. These findings are consistent with a previous study by Shemtov-Yona et al. who tested a conical 13 mm dental implant made of titanium alloy. Three different diameters (3.3, 3.75, and 5 mm) at the implant neck were tested for fatigue performance under cyclic loading. All 5 mm implants fractured at the abutment neck and screw, while all 3.3 and 3.75 mm implants fractured at the implant body. As the implants became thinner, they showed a tendency to fracture more apically than thicker samples [10]. While the results of this study showed no statistical difference between the fracture modes of two groups in the present study (*p* > 0.99), the one sample that fractured at the abutment level may have been affected by the diameter of the implant, and not solely from the design of the connection.

A limitation of this study is the relatively small size of the samples, three specimens for each group. While ISO 14801:2013 states that at least three specimens for each group is required, the small size of samples may not be enough to extract a general conclusion. However, the tendency of the results may provide a surmise on how different implant–abutment complexes react to fatigue. Also, another limitation of this study is that loading conditions such as the number of cycles, loading force, and loading angle were not similar to intraoral masticatory conditions. However, to the best of our knowledge, no testing apparatus or protocol currently can perfectly mimic the function of physiologic mastication. Additional research with large sample size and long-term cyclic loading program is required in the future. Also, a standardized testing protocol with further detail may be a prerequisite to the research. 

## 5. Conclusions

Within the limitation of this study, we conclude the following:

1. While both implant–abutment complexes are suitable for intraoral use, the EV fixtures in this study performed better than the TX fixtures, which indicates possible differentiation between the two implant–abutment complex designs.

2. Since all specimens with a 40% nominal peak value survived 5 × 10^6^ cycles and 50% specimens failed before 10^5^ cycles, a loading regimen with nominal peak value between 40% and 50% may be recommended for future testing of cyclic loading for the dental implant fixture.

3. The weakest parts of the tested fixtures were located at the first thread area, which happens to be the area directly coronal to the fixation simulating a 3 mm bone loss, and not necessarily the thinnest part. 

From these conclusions, future researchers and implant manufacturers may benefit from starting cycling loading at the loading regimen and by considering the weakest part presented when designing an implant. On the other hand, clinicians should consider the mechanical properties of the implants they plan to use.

## Figures and Tables

**Table 1:** Materials used in this study. All fixtures and abutments were composed of commercially pure grade 4 titanium.

Components	Test Group	Control Group
**Implant Fixture**	OsseoSpeed^®^ EV (4.2 mm × 11 mm)	OsseoSpeed^®^ TX (4.0 mm × 11 mm)
**Abutment**	TiDesign^®^ EV Abutment height: 5.5 mm Gingival height: 2.5 mm	TiDesign^®^ Abutment height: 5.5 mm Gingival height: 1.5 mm

Manufacturer: Dentsply Sirona Implants, Mölndal, Sweden.

**Table 2:** Values of the maximum breaking loads in single-load failure tests on three specimens each.

TX Ø4.0	Load at Break (N)	EV Ø4.2	Load at Break (N)
	698 N		856 N
	684 N		772 N
	752 N		745 N
**Mean ± SD**	711 ± 36 N	**Mean ± SD**	791 ± 58 N

**Table 3:** Values of the Fatigue Tests.

**TX Ø4.0**			
**Loading Level (%)**	**Sinusoidal Loading (N)**	**Number of Performed Cycles**	**Mean**
**80**	57–569	3209; 4369; 3851	3810
**60**	43–426	25,884; 14,353; 13,742	17,993
**50**	36–355	19,549; 66,014; 61,825	49,129
**40**	29–285	5,000,000; 5,000,000; 5,000,000	5,000,000
**EV Ø4.2**			
**Loading Level (%)**	**Sinusoidal Loading (N)**	**Number of Performed Cycles**	**Mean**
**80**	63–632	6696; 8567; 9333	8199
**60**	47–474	16,118; 39,423; 11,219	22,253
**50**	40–395	75,210; 23,584; 47,651	48,815
**40**	32–316	5,000,000; 5,000,000; 5,000,000	5,000,000

**Table 4:** Fisher’s exact test showed no difference between fractured areas (*P* > 0.99).

Fractured Area	Failure Aspect (TX Ø4.0)		Failure Aspect (EV Ø4.2)	
	Static Load	Cyclic Load	Static Load	Cyclic Load
**Abutment Fracture**	0	1	0	0
**Fixture Fracture**	3	8	3	9
